# Emotional dysregulation as a part of the autism spectrum continuum: a literature review from late childhood to adulthood

**DOI:** 10.3389/fpsyt.2023.1234518

**Published:** 2023-09-18

**Authors:** Liliana Dell’Osso, Leonardo Massoni, Simone Battaglini, Chiara De Felice, Benedetta Nardi, Giulia Amatori, Ivan Mirko Cremone, Barbara Carpita

**Affiliations:** Department of Clinical and Experimental Medicine, University of Pisa, Pisa, Italy

**Keywords:** emotional dysregulation, autism spectrum disorder, autistic traits, bioanatomical correlates, emotional regulation

## Abstract

The concept of emotional dysregulation (ED) has recently gained interest in the scientific literature and is commonly defined as the inability to use the modulatory mechanisms involved in emotion regulation, resulting in a functioning meaningfully below the baseline. Even though the data available are still limited, an increasing number of studies have hypothesized a promoting role for some of the core features of autism spectrum disorder (ASD) in the development of ED, in particular being repetitive behaviors, social difficulties and alexythimia. In this framework, the purpose of this study was to review the literature that is currently available about presence and correlates of ED in young adults with autism spectrum conditions as well as to offer some insights about possible implications for illness trajectories. The data reported seems to point to a shared etiology between ED and repetitive/restricted ASD symptoms, with perseveration features serving as the foundation for the inability to control one’s emotions. In this context, a neurodevelopmental basis for ED could be consistent with the transnosographic conceptualization of ASD, which hypothesizes a potential neurodevelopmental basis for several psychiatric disorders, whose autistic traits would be the phenotypical presentation.

## 1. Introduction

Autism Spectrum Disorder (ASD) is a heterogeneous neurodevelopmental disorder that can manifest with varying degrees of symptom severity, with or without intellectual impairment, and is characterized by a pervasive impairment in social and reciprocal communication and interactions, restricted and repetitive patterns of behavior, interests, or activities, a lack of socio-emotional reciprocity, and impaired sensory integration processing (1). Difficulties in socio-cognition are considered among the core symptoms of ASD, being at the basis of the deficits in social communication and interaction. In this framework, some authors also suggested that ASD deficits in affective empathy could be specific for the negative emotional valence while, on the other hand, subjects may still be able to empathize with the emotional experience of others when they express emotions with a positive valence (2, 3). Researches focusing on ASD have recently emphasized the need to examine not only the severe clinical forms but also the milder and sub-clinical manifestations, referred to as autistic traits (AT) which appear to be distributed along a continuum from the general to the clinical population (4, 5), and particularly represented in specific population such as students of scientific courses or psychiatric patients with other kinds of disorders (6–14). These traits can be classified in different dimensions, although research suggests that different dimensions may be associated to different outcomes (15, 16). In particular, subthreshold autistic features are of interest because they appear to have a negative impact on quality of life and to represent a major risk factor for the emergence of various psychiatric disorders, as well as suicidal thoughts and behaviors (17–19). The need for an early identification and intervention is therefore evident, since high intensity interventions have been reported to be able to reduce the majority of the main ASD symptoms (20). While in the psychiatric literature a growing interest has been paid to the concept of Emotional Dysregulation (ED), to date is still quite difficult to reach an agreement on ED definition. Despite the fact that the importance of “emotional problems” in clinical circumstances has led to an active search for causes, in which ED occupies a major place (21, 22), a common description of ED is still lacking. In 1991, Thompson defined emotion regulation as “a set of processes by which any individual assesses, inhibits, maintains, or modifies the intensity, frequency, or duration of emotional reactions in order to have the appropriate social behavior or to achieve goals” (23), while the concept of ED was proposed only few years later. To this date, one of the most welcomed definitions comes from Bunford, that defined ED as “an individual’s inability to exercise any or all aspects of the modulatory processes involved in emotion regulation, to such a degree that the inability results in the individual functioning meaningfully below his or her baseline” (24). It is now common opinion that ED is characterized by three main alterations: the first is an abnormal and excessive emotional response in comparison to social norms; the second is uncontrollable and rapid fluctuations in emotions; and the third is an aberrant allocation of attention to emotional stimuli (25). Moreover, ED comprehends the inability to detect and accept emotions as well as a lack of ability to adaptively select efficient coping mechanisms (26–28). According to an increasing number of studies, the core features of autism spectrum may be at the basis of the development of ED, the latter being particularly associated with repetitive behaviors, social difficulties and alexythimia as promoting factors (29–31). In this framework, ED may be considered one of the steps of the illness trajectory that, from the presence of autistic traits, often lead to the development of mood disorders (30–33). This issue is of particular importance considering the increased prevalence of autistic traits in different kinds of psychiatric conditions, to the point that some authors supported a possible neurodevelopmental approach to mental disorders, with autistic traits conceptualized as a trans-nosographic dimension (34–37). According to this model the specific timing, localization and severity of neurodevelopmental alterations, interacting with environmental factors and life events, may shape specific illness trajectories in different subjects (34–36).

Moreover, in recent years, there has been substantial attention in the relationship between ED and non-suicide self-injury, particularly considering that non suicidal self harm rates have been growing and they are considered a critical risk factor for suicidal behavior (38, 39). Two main models have been proposed as possible explanations for this link. The experiential avoidance model of deliberate self-harm, makes the case that non-suicide self-injury fulfills the function of avoidance or escape from undesirable emotional arousal (40). While, similarly, the emotional cascade model proposes a clear link between ED and dysregulated behaviors (such as self harming) suggesting that rumination, which is, on the other hand, often underlain by autistic traits, increases emotional experiences, causing people to engage in dysregulated activities as a temporary distraction from negative emotion (41).

Several different scales have been proposed to measure ED and are applicable in ASD individuals. Those include the Emotion Dysegulation Inventory (EDI), that consists of 66 items, the Aberrant Behavior Checklist (ABC), that takes into account irritability, lethargy/social withdrawal, stereotypic behavior, hyperactivity and inappropriate speech, and the Child Behavior Checklist (CBCL), that includes four domains: *Anxious/Depressed* (13 items), *Withdrawn/Depressed* (8 items), and *Aggressive Behavior* (18 items) (42). In this framework, many studies searched for the bio-neurological correlates of ED in ASD subjects, hypothesizing a role for some dopaminergic gene polymorphisms that may promote ED in children with ASD (43), and reporting a correlation between specific brain regions, including middle frontal gyrus (MFG), posterior insula (PI), amygdala and prefrontal cortex (PFC) and problem in emotional regulation in ASD subjects (30, 44).

In this framework, the aim of this work was to provide a narrative review of the available literature about presence and correlates of ED in adolescents and young adults with autism spectrum conditions, providing also some insights about possible implications for illness trajectories. For this purpose, articles were searched on two electronic databases (PubMed and Scopus) from January 26th to February 14th 2023. The following search terms, without filters, restriction or limits, were used to identify all potentially eligible records: (“emotional dysregulation” OR “affective dysregulation” OR “emotion dysregulation”) AND (“neurodevelopmental disorders” OR “autism” OR “ASD” OR “autism spectrum disorder”). Papers were screened separately by reviewers for inclusion and disagreements were resolved by discussion. Because we aimed to investigate the prevalence of ED in human subjects, studies investigating ED in animal models were excluded. Moreover, reviews, case reports and editorials were also excluded due to the nature of the articles.

## 2. ED in ASD: from adolescence to adulthood

### 2.1. Emotional regulation strategies

ED is reported to be a frequent feature of ASD that is often present from the earliest ages. A study conducted by Samson et al. (45) investigated a wide range of emotion regulation techniques in a sample of 32 ASD children and adolescents, diagnosed according to the Diagnostic and Statistical Manual (DSM) IV-TR criteria (*F* = 3, M = 29, age range = 8–20 years) and 31 typically developing (TD) participants (*F* = 8, M = 23, age range 8–20 years). Parents of both groups were interviewed about their child’s emotional experience and the use and efficacy of 10 ER strategies and answered the Social Responsiveness Scale (SRS), the CBCL and the Stanford Binet for evaluating cognitive function. Results highlighted that, when asked about their children’s behaviors, ASD parents reported a reduced use of adaptive strategies (problem solving, cognitive reappraisal) and increased of maladaptive ones (repetitive behaviors) in comparison to TD parents (45). The same authors also investigated emotional regulation in 21 ASD children and adolescents diagnosed through the DSM-IV criteria (M = 18, *F* = 3, age range = 8–20 years) and 22 matched TD controls (M = 16, *F* = 6, age range 8–20 years). The two groups were evaluated for cognitive functions through the Full Scale Intelligence Quotient (FSIQ), cognitive function was assessed using Stanford Binet and emotional reactivity and regulation in children and adolescents with and without ASD was assessed through the Reactivity and Regulation Situation Task (RRST). Additionally, TD subjects were screened via face-to-face evaluations, telephone interviews and observation during psychometric tests. According to the findings, individuals with ASD showed the same level of reactivity to negative stimuli as TD participants. Furthermore, ASD adolescents presented a different emotion regulation profile than TD individuals, characterized by a less frequent use of cognitive reappraisal and more frequent use of suppression. In addition, when instructed to use cognitive reappraisal, ASD participants showed difficulties to use it, but benefitted from this strategy when they were able to generate a reappraisal (46). Results from both studies prompted the authors to suggest that ED could be considered a core feature of ASD and that the development of treatment programs that focus on improving the use of adaptive forms of emotion regulation may reduce emotional difficulties and improve long-term results in children and adolescents with ASD.

Another study (29) compared a group of 56 ASD individuals (M = 47, *F* = 9, age range = 6–16 years) with 38 TD controls (M = 26, *F* = 12, age range = 6–16 years). Participants were assessed for ASD core features through: the Stanford Binet 5^th^ edition (SB5), to determine cognitive functioning; the Vineland Adaptive Behavior Scales, 2nd Edition (VABS-2) to measure adaptive behavioral functioning in the domains of Socialization, Communication, Daily Living Skills, Motor Skills, and an overall Adaptive Behavior Composite score; the SRS, to investigate Social Cognition, Social Motivation, Social Awareness, Social Communication, and Autism Mannerisms; the Repetitive Behavior Scale-Revised (RBS-R), to capture the domains of Stereotyped Behavior, Self-Injurious Behavior, Compulsive Behavior, Ritualistic Behavior, Sameness Behavior and Restricted Behavior; the Short Sensory Profile (SSP) to provide subscales that reflect atypical sensory processing in different domains (Tactile Sensitivity, Taste/Smell Sensitivity, Movement Sensitivity, Underresponsive/Seeks Sensation, Auditory Filtering, Low Energy/Weak, and Visual/Auditory Sensitivity) and a total score. The emotional deregulation index was provided through the CBCL, a parent-report measure assessing problem behaviors (e.g., aggressive behavior). Results reported that ED was related to all core features of the ASD, but interestingly the strongest association was with repetitive behaviors which appeared to be the best predictors of ED. This finding could imply that people with ASD who have significant repetitive and restricted symptoms are less able to regulate their emotions due to difficulties inhibiting ongoing behaviors. This finding is congruent with the findings of Mazefsky et al. (47), who proposed that perseveration (an example of a repetitive action) can lead to the development and maintenance of emotion control issues. However, another possibility is that ED in ASD activates compensatory control systems, which manifest as restricted and repetitive behaviors.

A further research studied the impact of different emotion regulation strategies among subjects with ASD (48). The author focused on reappraisal, an emotion regulation strategy based on re-interpreting a situation in order to change the way one feels about it. Another evaluated strategy was suppression, which features the inhibition of emotional expression. The authors enrolled 56 ASD adolescents and young adults (M = 39, *F* = 17, age range = 14–24 years), who were divided into 4 groups according to Emotion Regulation Questionnaire subscale scores. It was found that individuals in the high suppression and low reappraisal group showed higher depressive symptoms and reduced well-being compared with subjects in the low suppression and high reappraisal group. Meanwhile, those who reported to use both high suppression and reappraisal expressed relatively high positive well-being and low depression symptoms. It was speculated that the habitual use of reappraisal may buffer the effect of regular suppression usage (48).

### 2.2. Relationship between ED and ASD core features

A work by Neuhaus et al. (49) explored the role of social skills as a mediator of ED in ASD. A group of 2079 individuals (M = 1803, *F* = 276, age range = 72–216 months) were evaluated for ED, social functioning, and cognitive skills. The authors reported direct effects of social motivation, internalizing symptoms, aggression, attention problems, irritability, and self-injurious behavior on children’s social skills. In addition, ED moderated the association between social motivation and social skills. Globally, these results not only identified risks for poorer social and emotional outcomes in children and adolescents with ASD, but also suggest areas for intervention. Whereas the main effects of dysregulation on social skills indicate a clear need to intervene in emotion-related processes to facilitate social functioning, the moderating role of ED on social functioning suggests that such support may be beneficial even when dysregulation difficulties are not readily apparent (49).

A more recent study from Patel et al. (50), aimed to investigate the link between ED and other symptoms frequently related to ASD, such as anger rumination and depression. For this purpose, a sample of 25 ASD adolescents and 24 controls (age range 12–19 years) was recruited and they were all evaluated with the Anger Rumination Scale (ARS) to assess feelings of anger, the SRS and the Mood and Feelings Questionnaire (SMFQ) for depressive symptomatology. Results suggested that anger rumination was related to the presence of depression and general emotional and behavioral dysregulation in individuals with ASD (50). Taken together, these findings are in line with previous researches (47) thesis that the proclivity to persevere in ASD includes recurrent and persistent thoughts regarding negative emotional events, such as anger/frustration and depression symptoms. Moreover, the relationship between anger rumination and worse emotional and behavioral control in non-ASD samples is consistent with the emotional cascade model of behavioral dysregulation, which proposes that rumination enhances negative affect, which can then lead to dysregulated behaviors (51). Thus, treatment strategies to minimize ruminating in ASD may be beneficial in alleviating a variety of symptoms. In a pilot study of people with ASD, for example, a mindfulness intervention reduced both rumination and mood symptoms (52). If indicated, intervention addressing ruminating may be especially significant because those who ruminate tend to do so indefinitely (53).

When discussing the relationship between core symptoms of ASD and ED, one major goal has been the development of a useful as a psychometric tool, such as the Difficulties with Emotion Regulation Scale (DERS), a scale that contains 36 items rated on a 5-point Likert scale ranging from 1 (almost never) to 5 (almost always). A study on the psychometric properties of DERS, conducted by McVey et al. (54) analyzed datas from 4 different studies collecting a total of 156 ASD adolescents and adults. Inclusion criteria were 1) having item-level data on the DERS and 2) being classified as autistic in their respective studies. Results highlighted that DERS was positively associated with anxiety, depression and alexithymia, thus suggesting the usefulness to test DERS in autistic population (54).

### 2.3. ED and intolerance of uncertainty

Furthermore, a recent interest has been posed to the role of ED and intolerance of uncertainty (IU), described as the “inability to tolerate the unwanted cognitive, emotional and behavioral effects triggered by a perceived lack of information for a given situation” (55) in the ASD population. A study by Saez-Suanes et al. (56) shed new light on the role of ED and UI as mediators of anxiety in ASD. A sample of 121 individuals (M = 81, *F* = 40, age range 18–62 years), with a diagnosis of ASD and intellectual disability (ID), were evaluated to determine the predictive and mediating role of executive functioning, emotional regulation and IU, by using the Diagnostic Behavioral Assessment for Autism Spectrum Disorder-Revised (DiBAS-R), the Anxiety Scale for Adults with ASD (ASA-ASD-I), the Emotional Regulation Checklist (ERC) and the Intolerance of Uncertainty Scale – Parent Version (IUS-12-P). Results showed that IU was a predictor of anxiety, and supported the mediating role of UI and emotional regulation between ASD and anxiety, thus suggesting that interventions designed to reduce anxiety symptoms in people with ASD and ID should include among their goals emotional regulation and especially improvement in intolerance of uncertainty (56). In addition, a study from Conner et al. (57) evaluated 78 adolescents and young adults (M = 61, *F* = 15, transgender = 2, age range = 12–20 years) through the Intolerance of Uncertainty Scale (IUS-12), the PROMIS anxiety and depression scales and the EDI. In particular, the latter is one of the scales most commonly used to measure ED in ASD and structured in a caregiver-report questionnaire designed to capture emotional distress and problems with emotion regulation without requiring verbal information (37). The authors concluded that ED contributes to how IU affects psychopathology, specifically, ED, IU, anxiety, and depression scores were all correlated with each other. Additionally, both Reactivity and Dysphoria were found to mediate IU-depression and IU-anxiety (58). Overall, these findings are consistent with the rising body of literature indicating the extensive role of ED and IU in ASD psychopathology (59). Furthermore, they add to the growing body of literature highlighting the significance of IU as a transdiagnostic construct related with different kinds of psychopathology in ASD (59–61), as well as its potential as a transdiagnostic, trans-therapy changes process (62).

### 2.4. ED and alexithymia

Some authors examined the role of ED together with interoceptive sensibility and alexithymia, defined as difficulties in identifying and verbalizing emotions, in somatoform disorder in ASD. Zdankiewicz-Ścigała et al. (63) compared 79 ASD individuals (M = 22, *F* = 57%, age range 20–45 years) and 126 controls (M = 26, *F* = 100, age range = 18–63 years). Participants were evaluated through The Toronto Alexithymia Scale–20 (TAS-20), the Autism-Spectrum Quotient (AQ) and the Difficulties in Emotion Regulation Scale (DERS). Results showed a positive correlation between interoceptive sensibility and alexithymia, and ED in the lack of emotional awareness and lack of emotional clarity (63). The role of alexithymia together with ED as a mediator between anxiety /depression and autistic symptoms was also explored by Morie et al. (30), who analyzed 64 ASD individuals (M = 17, *F* = 47, age range = 18–65 years) through the SRS, Depression, Anxiety and Stress Scale (DASS), TAS-20 and DERS. It was found that the autistic symptoms mediated both y anxiety and depression through a pathway featuring alexithymia and ED as intermediate steps. As a result, targeting alexithymia may benefit therapies designed to alleviate mood disorders in ASD (30). Gormley et al. (31) reported similar findings in a work carried out in 43 ASD subjects (M = 32, *F* = 11, age range = 10–18 years) assessed through self and parent reported questionnaires such as the Alexithymia Questionnaire for Children (AQC), Emotion Regulation Index for Children and Adolescents (ERICA), Emotion Regulation Checklist (ERC) and Autism Spectrum Rating Scale (6–18 years Parent Ratings) (ASRS). High levels of alexithymia and ED were reported by both patients and their parents. Moreover, alexithymia significantly correlated with emotion regulation (31). These findings are consistent with previous studies that established a link between alexithymia and interoception in patients with ASD. For instance, Gaigg et al. (64) established that alexithymia is caused by a disturbance in how physiological arousal regulates subjective sensations in people with and without ASD. The findings support the validity of models that consider physiological arousal perception (interoception) to be crucial in the subjective experience of feelings (65–67). Subjects with alexithymia may either fail to recognize normally normal physiological arousal or exhibit atypical arousal, which has an impact on their subjective emotional experience (68).

Another interesting study compared 60 ASD individuals (M = 30, *F* = 30, mean age = 14 years), 60 adolescents with Attention Deficit Hyperactivity Disorder (ADHD) (M = 30, F = 30, mean age = 14 years) and 60 controls (M = 30, F = 30, mean age = 13.55 years) for ED aspects (69). Reading the mind in the eyes test, the Faux Pas Test, and the hinting task were given to patients to evaluate the Theory of Mind (ToM) skills. Results pointed out that all these individuals had defects in emotional regulation, with ASD subjects presenting more difficulties than the ADHD ones (69).

## 3. Bio-anatomical correlates of ED in ASD

From a biochemical point of view, the available literature reported the involvement of some dopaminergic gene polymorphisms in the modulation of ED in children with ASD (43) as well as its correlation with specific brain regions. In particular, a work by Ibraihim et al. (70) compared three different groups of children (age range = 8–16 years): ASD-plus-Disruptive-Behavior (ASD + DB) (*n* = 18), ASD without disruptive behavior (ASD) (*n* = 20) and TD (*n* = 19). ASD was diagnosed through the DSM-5, and participants were evaluated with the K-SADS-PL. Parents were invited to complete the CBCL and the Inventory of Callous-Unemotional Traits (ICU). Participants were also examined through a functional magnetic resonance imaging (fMRI), and completed an emotion perception task of fearful vs. calm faces. The authors analyzed task-specific changes in amygdala reactivity and connectivity by using whole-brain, psychophysiological interaction, and multiple-regression analyses and concluded that reduced amygdala-vIPFC connectivity during fear processing may differentiate children with ASD + DB from children with only ASD. In addition, they revealed that the presence of callous-unemotional traits could be useful for identifying patterns of amygdala activity associated with increased risk of aggression in ASD patients. These findings suggested a neural mechanism of ED associated with disruptive behavior in children with ASD (70). In order to better investigate the neurostructural correlates of ED in ASD, Ni et al. (71) acquired diffusion spectrum imaging in two groups: 59 ASD children (mean age = 12.5 years) and 62 TD (mean age = 12 years). The relationship between participants’ dysregulation levels and microstructural property of 76 white matter (WM) tracts was evaluated in a multivariate analysis across diagnostic groups concluding that both ASD and TD individuals had a broad distinct WM microstructural property underpinning self-regulation (71). Another study analyzing the mechanisms of sustained processing of negative information in brain activity in ASD by using fMRI, enrolled 25 ASD adolescents (M = 24, *F* = 1, mean age = 14.95 years) and 23 TD (M = 22, F = 1, mean age 15.5 years). Results highlighted that the salience network and the prefrontal dorsolateral cortex, which previous studies also reported to be implicated in ED, were the brain regions identified as having greater and more continued processing following negative stimuli in the ASD group compared to the TD group. Interestingly, there was also an association between brain activity in the identified regions and parent-reported ED in ASD (72).

Moreover, it should be noted that ED has also been associated with autonomic nervous system deregulation, another typical feature of ASD (73). Poor emotional regulation in ASD is knowingly associated with physiological arousal, negative and positive effects, alteration in the amygdala and prefrontal cortex (74). Furthermore, irritability in ASD boys has been associated with physiological reactivity, as reported in a study from Mikita et al. (75). The sample consisted in 47 high functioning male ASD (HF-ASD) (mean age = 12.8 years) and 23 TD subjects (mean age = 13.9 years), who completed a psychosocial stress test. The variables recorded were: changes in cortisol, heart rate and heart rate variability throughout the test as well as self- and parent-reported measures of irritability. Irritability symptoms reported in the HF-ASD group were compared to two groups of subjects without ASD: 40 highly irritable boys or severe mood dysregulation (SMD) group and 30 healthy-control (HC). Results reported that the HF-ASD group had higher score on irritability than the HC, together with a pattern of irritability symptoms closely resembling that of the SMD group. Even though boys with HF-ASD presented significant stress-induced changes in cortisol and heart rate, a lower cortisol level throughout the test was found in those who rated themselves as highly irritable compared to those low on irritability. Blunted cortisol and heart rate responses to stress emerged in participants rated as highly irritable by their parents. The effects of irritability on heart rate, but not cortisol, were accounted for by trait anxiety (75). Another nervous system variable examined in ASD emotional deregulation is heart rate variability (HRV). A work was carried out in 23 ASD individuals (M = 16, *F* = 7) and 32 TD controls (M = 8, *F* = 24). Participants, aged between 13 and 18 years, were assessed for ED through the EDI inventory, while a short-term electrocardiogram was used for HRV. It was found that lower resting HRV was strongly associated with greater ED in ASD than in TD controls, thus suggesting that alterations of autonomic functioning may contribute to emotion dysregulation in ASD (76).

## 4. Therapeutic perspectives in ASD

Non-pharmacological approaches to the treatment of ED in ASD include various meaningful therapeutic strategies. Of primary importance is an evidence-based practice, namely the use of functional communication training, which systematically teaches a functional equivalent to the maladaptive behavior (77). Another therapeutic strategy was presented by Torrado et al. (78) and consists in two devices: a smartwatch that detects the user’s inner state and displays the self-regulation strategies and a smartphone, used by caregivers or family members in an adaptive way to create, edit and customize such interventions. The smartwatch monitors the user’s heart rate and infers outburst patterns from physiological signals and movements in order to be able to manage a lot of minor outbursts. The behavior’s long-term issues are based on repeated episodes of exposure to unpleasant, self-harming sensations not properly addressed due to. According to the authors, this system may allow a major improvement on the quality of life of people with ASD and ED problems, considering that small-scale intervention applied to the daily life of these individuals seems to prevent long-term behavioral issues (78). More specific therapeutic approaches, such as cognitive behavioral therapy (CBT) is often used with patients with ASD in comorbidity with mood disorder, anxiety, and/or behavioral difficulties that have at least average verbal capabilities (77). In this framework, a study conducted by Bemmouna et al. (79) valuated the feasibility, acceptability and preliminary efficacy of Dialectical Behavior Therapy (DBT) in seven ASD adults without ID exhibiting self-harm and/or suicidal behaviors linked to severe ED (age range = 19–56, mean age = 27.71 ± 13.34) assessed with the DERS. DERS total scores ranged from 107 to 144 (mean score = 123.57 ± 13.56). The results showed that mean scores decreased significantly post-treatment and at 4-month follow-up, suggesting that DBT might be efficacious in reducing ED in this population. The majority of participant also showed a decrease in the frequency and severity of self-harm and reported a decline in the frequency of suicidal thoughts (79). Another promising research by Huntjens et al. (80) aimed to evaluate the efficacy of DBT in ASD subjects and its influence on suicidal and self-destructive behaviors. For this purpose, 128 subjects with autism and suicidal and/or self-harming behavior were recruited and divided in two groups, one where the participants have weekly individual cognitive behavioral therapy sessions and a 2.5 h skills training group session twice per week during 6 months and another where subjects are treated with weekly individual therapy sessions of 30–45 min with a psychotherapist or social worker. To this date, the study is ongoing and in the data collection stage, however results from this study could be promising in giving an evaluation of the efficacy of DBT treatment in persons with ASD on suicidal and self-harming behavior (80).

## 5. Discussion

Overall, a limited number of studies focused on ED in ASD from adolescence to adulthood, while more data are available on child or mixed samples. ASD individuals have been reported to show lower adaptive behaviors, less use of cognitive reappraisal and more use of emotional suppression than TD controls (46) and even ADHD patients (69). Additionally, the scientific literature highlighted difficulties in identifying and verbalizing emotions with lack of emotional awareness and clarity in ASD (63). Besides, ASD people tend to present more anger, rumination and depression than controls (50). According to some authors, ED and IU seem to be related to increased depression and anxiety levels in ASD, possibly influencing each other (57, 66). Noticeably, ED in ASD has been related with specific genetic polymorphisms (43) as well as with anatomical alteration such as reduced amygdala connectivity (70). Another brain region implicated in ED is the prefrontal dorsolateral cortex (72) while, together with genetic and anatomical alterations, other studies reported autonomic nervous system deregulation, such as an alteration in cortisol levels and heart rate, in ASD individuals (73, 75). Intriguingly, the same autonomic nervous system deregulation seems to promote ED according to another study (76), possibly suggesting that the development of ED could be linked to the underlying ASD condition also from a neurophysiological point of view (76). In this framework, it should be noted that, besides stressing a frequent presence of ED among subject with ASD, some authors are also hypothesizing a link between the core features of autism spectrum and ED: ED was reported indeed to be linked with social difficulties and, in particular, with the presence of repetitive behaviors and restricted interest (29). This data may suggest a common pathophysiology between ED and repetitive/restricted symptoms of ASD, with perseveration traits at the basis of the difficulties in regulating emotions (29). A neurodevelopmental basis for ED could be in line with the transnosographic conceptualization of autism spectrum (39), which, on the basis of the frequent presence of autistic traits in different psychopathological conditions, hypothesizes a possible neurodevelopmental underpinning (whose autistic traits would be the phenotypical presentation) for several psychiatric disorders (40). The presence of a neurodevelopmental alteration would also feature a vulnerability towards life events, facilitating the development of trauma and stress related symptoms as well as a reduced ability to regulate emotions (40). This hypothesis seems to be further supported by the findings of Morie et al. (30), which reported a mediating pathway that, starting from the presence of autistic traits, leads, through the development of alexithymia and ED, to full-blown anxiety and mood disorders. This data is somewhat in line with another study by Dell’Osso et al. (32) which reported a significant effect of autistic traits in enhancing the development of mood disorders, directly and indirectly, through the effect of post-traumatic symptoms (often associated in the literature with ED) and rumination (32).

Globally, despite the limited literature available, some interesting evidences on the prevalence of ED in ASD, as well as on its bio-neurological correlates, were highlighted. However, many aspects of this dimension remain controversial and should be further investigated. In particular, further studies are needed for evaluating ED, in light of a neurodevelopmental approach to psychopathology, as a possible step of the pathway that from autistic traits may promote the development of other psychiatric conditions.

## Author contributions

LD’O, IMC, and BC: conceptualization. LD’O, IMC, and BC: methodology. LM, SB, and CDF: investigation. LM, SB, CDF, GA, and BN: writing—original draft preparation. BC, GA, and BN: writing—review and editing. LD’O, BC, and IMC: supervision. All authors contributed to the article and approved the submitted version.
